# Position preference of essential genes in prokaryotic operons

**DOI:** 10.1371/journal.pone.0250380

**Published:** 2021-04-22

**Authors:** Tao Liu, Hao Luo, Feng Gao

**Affiliations:** 1 Department of Physics, School of Science, Tianjin University, Tianjin, China; 2 Frontiers Science Center for Synthetic Biology and Key Laboratory of Systems Bioengineering (Ministry of Education), Tianjin University, Tianjin, China; 3 SynBio Research Platform, Collaborative Innovation Center of Chemical Science and Engineering (Tianjin), Tianjin, China; Huazhong University of Science and Technology, CHINA

## Abstract

Essential genes, which form the basis of life activities, are crucial for the survival of organisms. Essential genes tend to be located in operons, but how they are distributed in operons is still unclear for most prokaryotes. In order to clarify the general rule of position preference of essential genes in operons, an index of the average position of genes in an operon was proposed, and the distributions of essential and non-essential genes in operons in 51 bacterial genomes and two archaeal genomes were analyzed based on this new index. Consequently, essential genes were found to preferentially occupy the front positions of the operons, which tend to be expressed at higher levels.

## Introduction

Essential genes usually refer to genes whose inactivation or loss causes either severe growth impairment, irreversible growth arrest, or cell death [1]. Essential genes are necessary for cells or organisms to survive under specific conditions [2, 3]. These genes constitute the minimal gene set required for living cells. Therefore, the functions encoded by this gene set are considered the basis of life [4, 5]. The study of essential genes has become a hot topic, as it is helpful to explore the origin and evolution of life, as well as provide an important basis for discovery of drug targets [6, 7], treatment of diseases [1, 8], and design of minimal genomes [9, 10]. Currently, essential genes can be identified through a series of experimental methods, including transposon mutagenesis [11], antisense RNA silencing [12], single-gene knockout technology [13], and other methods. An increasing number of essential genes have been genome-widely identified, and this facilitates the study of characteristic differences between essential and non-essential genes. For example, in prokaryotes, essential genes are found to be preferentially located on the leading strand of chromosomes [14, 15], and further studies have shown that only those with certain COG functional subclasses are preferentially located on the leading strand [16, 17]. Proteins corresponding to essential genes were enriched in the cytoplasm, and the proportion of non-essential genes in the plasma membrane, periplasm, outer membrane, cell wall, and extracellular space is significantly higher than that of essential genes [18]. Essential genes in genomic islands are significantly fewer than those outside of genomic islands [19]. Compared with non-essential genes, bacterial essential genes tend to encode core functions related to transcription, translation and replication [4, 20], and have a higher ratio of enzymes [21]. In addition, essential genes have higher expression levels than non-essential genes [22, 23] and are more evolutionarily conserved [24, 25].

An *operon* is the set of one or several genes and their associated regulatory elements, which are transcribed as a polycistronic unit [26, 27]. Operons are widely used as basic transcriptional and functional units [28]. Regarding operon formation, the most widely accepted theory is the co-regulation hypothesis, which assumes that operons are formed by rearranging two or more genes together, while maintaining this structure by selecting a coordinated transcriptional regulation and translation of functionally related proteins [29, 30]. Regarding the evolution of operons, the regulatory model and selfish model are two generally accepted models [31]. The former emphasizes the advantage of co-transcription for regulatory purposes, while the latter emphasizes the advantage of genome proximity for co-transfer of adjacent functions [32]. Other proposed operon evolution models have received less attention, mainly because they do not conform to the existing evidence [33]. According to the co-regulation hypothesis, essential genes are preferentially located in operons, which has been confirmed in *Escherichia coli* [29, 30, 34]. In addition, studies have found that essential genes are not only preferentially located in operons, but also often occupy the first position in operons [35]. However, this research has certain limitations, such as the relatively small number of prokaryotic genomes analyzed, and conclusions drawn without considering the influence of the proportion of essential genes in an operon on which gene occupies the first position. In particular, focusing only on the preference of the first operon position does not lead to a general conclusion on the position preference of essential genes in operons.

With the wide application of high-throughput experimental technologies in the identification of essential genes, essential genes data has increased rapidly, and the essential genes database DEG is also constantly updated to include these essential genes data. However, at present, the distribution of essential genes in most prokaryotic operons listed in DEG 15 is not clear. As reliable information in the operons database becomes available for more prokaryotic genomes, a systematic study on the distribution of essential genes in operons in prokaryotic genomes is possible.

In the present work, the preferences of essential and non-essential genes for special positions in operons were studied for 53 prokaryotic genomes, including 51 bacteria and 2 archaea. By analyzing the distribution of essential genes in operons, it was found that essential genes preferentially occupy the first position of operons, as reported in a previous study. However, after removing operons in which all genes are essential genes, the rule becomes invalid. Here, an index of the average position of genes in an operon is proposed to measure the position preference of essential genes in operons. By comparing the average positions of essential and non-essential genes in operons, it was found that essential genes tend to occupy the front positions of operons compared to non-essential genes, which was also confirmed by analyzing the proportion of essential genes located in the first half of operons.

## Materials and methods

### Data source

The essential genes data of the 53 prokaryotic genomes studied here were downloaded from the DEG database (version 15) [36] (http://essentialgene.org/). For some genomes, essential genes have been identified through different experimental methods. In this study, only one essential genes set was reserved by considering the reliability of the method used or the results. The corresponding operons data were obtained from the DOOR database [28] (http://161.117.81.224/DOOR3). For the prokaryotic genome with multiple chromosomes, only the essential genes on the main chromosome were studied. For the operons data in the DOOR database, only multi-gene operons were regarded as operons.

### Determination of DNA strands

The replication origins and termini were derived from the DoriC database [37, 38] (http://tubic.tju.edu.cn/doric/), based on which the leading and lagging strands for each genome can be determined.

### Index of average position of genes in an operon

Assuming that an operon contains *n* genes, including *n*_1_ essential genes and *n*_2_ non-essential genes (1≤*n*_1_<*n*, 1≤*n*_2_<*n*), the position occupied by a certain gene is *x*, and the average position of genes in an operon is defined as

$$\bar{\text{X}}=\frac{\sum_{\text{i}=1}^{n}x_{i}}{n}=\frac{1+2+\ldots +n}{n}=\frac{n+1}{2}.$$ (1)

Similarly, the average position of essential genes in an operon is

$${\bar{\text{X}}}_{\text{EG}}=\frac{\sum_{i=1}^{n_{1}}x_{i}^{EG}}{n_{1}}.$$ (2)

And the average position of non-essential genes in an operon is

$${\bar{\text{X}}}_{\text{NEG}}=\frac{\sum_{j=1}^{n_{2}}x_{j}^{NEG}}{n_{2}}.$$ (3)

And the relative position of essential genes in an operon is calculated as follows:

$${\text{D}}_{\text{EG}}={\bar{\text{X}}}_{\text{EG}}-\bar{\text{X}}$$ (4)

Only operons containing at least one essential gene were considered. It should be noted that if all the genes in an operon are essential genes, the position is all occupied by an essential gene. Therefore, only the positions in operons in which both essential and non-essential genes exist were analyzed.

## Results and discussion

### Position preference of essential genes in operons

#### Position preference of essential and non-essential genes in special positions of operons

Essential genes in *E*. *coli* have been found to be enriched in operons [39], but whether this is a common feature of other bacteria and archaea needs to be verified. There was a clear trend for essential genes to occupy operons across 44 prokaryotic genomes (*P* ≤ 0.05, Fisher’s exact test) (S1 Table in S1 File). Further, the statistical significance was very high in 33 of these conditions (*P* < 2.0 × 10^−4^, Fisher’s exact test) (S1 Table in S1 File).

**Fig 1:**
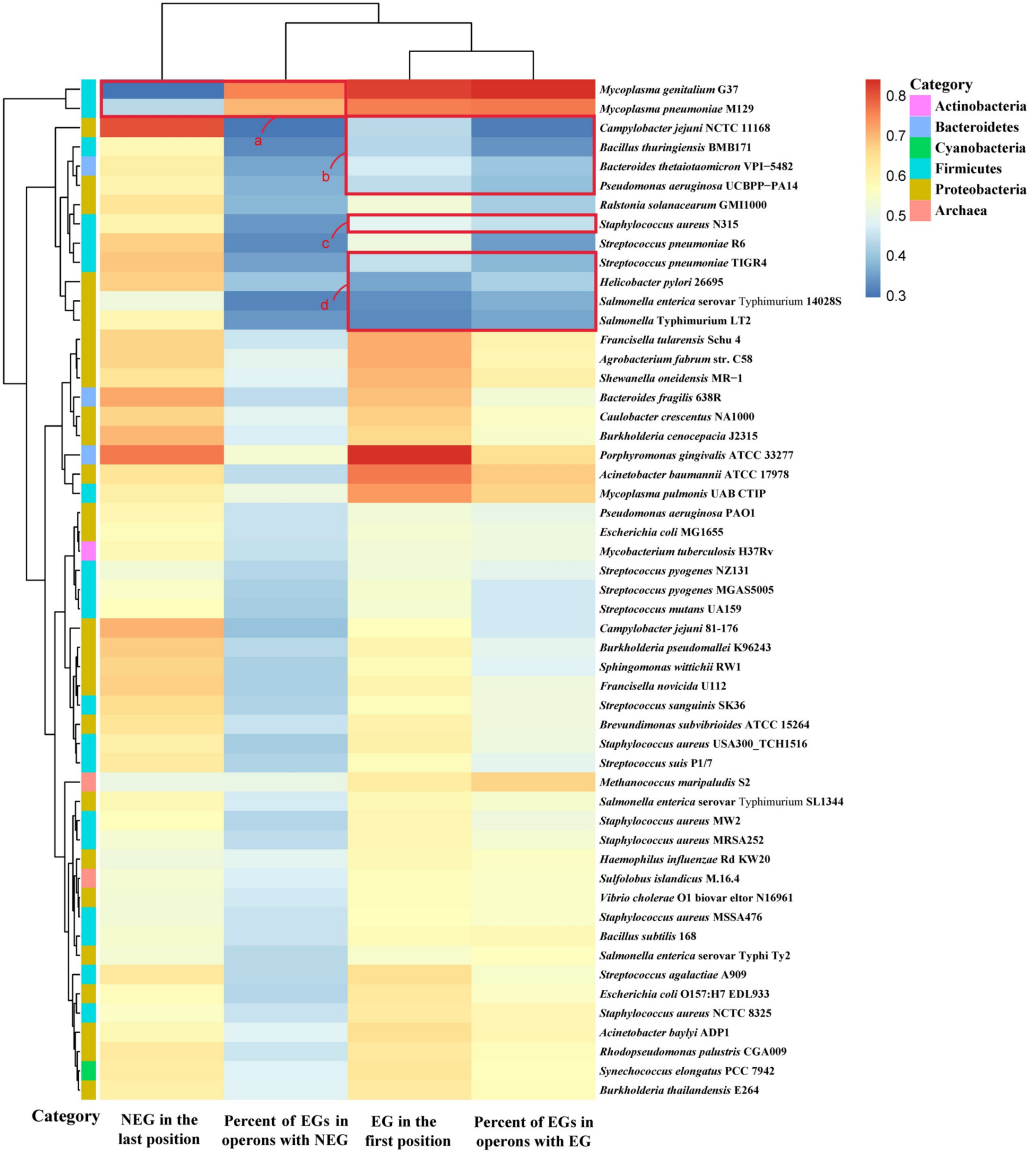
The relationship between distribution of essential and non-essential genes and proportion of essential genes. The heatmap was plotted using the heatmap function in the R package. The cells in the heatmap correspond to the proportion of genes under different conditions, and the value range is displayed in different colors. The color bar on the left side of the heatmap corresponds to the phylum classification of the species. Hierarchical clustering of analysis results in two dimensions is represented by a tree diagram. Species whose distribution of essential genes occupies the first position in less than 50% of operons are shown in red square boxes b-d, and species whose distribution of non-essential genes occupies the last position in less than 50% of operons are shown in red square box a.

It was also found that most of the essential genes preferentially occupied the first position of the operon they were located in (Fig 1). Among them, in 44 genomes, there are more than 50% of operons in which the essential genes occupy the first position (S2 Table in S1 File), consistent with previous results. Among 39 genomes, compared with non-essential genes, essential genes tend to occupy the first position of the operon (*P* ≤ 0.05, Fisher’s exact test) (S2 Table in S1 File). We also studied the distribution of essential genes in operons containing two and three genes, and performed a chi-squared test, which confirmed that essential genes preferentially occupy the first position in operons of most species (*P* ≤ 0.05; S3 Table in S1 File). In addition, the distribution of non-essential genes in the operons was analyzed. Consequently, in 53 prokaryotic genomes, non-essential genes were found to frequently occupy the last position of the operon (Fig 1). Among them, in 51 genomes, in more than 50% of operons, non-essential genes occupy the last position (S2 Table in S1 File). In 37 genomes, compared with essential genes, non-essential genes tend to occupy the last position of the operon (*P* ≤ 0.05, Fisher’s exact test) (S2 Table in S1 File).

We found that the positions occupied by essential and non-essential genes were related to the proportion of essential genes out of all the genes in operons (Fig 1). As can be seen from Fig 1, the essential genes of *Mycoplasma genitalium* G37 and *Mycoplasma pneumoniae* M129 account for a higher proportion of the genes in operons, resulting in a lower proportion of non-essential genes occupying the last position of the operon (box a in Fig 1). The essential genes of *Staphylococcus aureus* N315, *Bacteroides thetaiotaomicron* VPI-5482, *Streptococcus pneumoniae* TIGR4, *Pseudomonas aeruginosa* UCBPP-PA14, *Campylobacter jejuni* NCTC 11168, *Bacillus thuringiensis* BMB171, *Helicobacter pylori* 26695, *Salmonella enterica* serovar Typhimurium 14028S, and *Salmonella* Typhimurium LT2 account for a low proportion of the genes in operons, resulting in a low proportion of essential genes occupying the first position of operons (boxes b-d in Fig 1). The Pearson correlation coefficient [40] between the proportion of essential genes occupying the first position of operons and the proportion of essential genes in operons was 0.88, while the Pearson correlation coefficient between the proportion of non-essential genes occupying the last position of operons and the proportion of essential genes in operons was −0.52. From these 53 prokaryotic genomes, the rule can be summarized as follows: the higher the proportion of essential genes in the genes in operons, the higher the proportion of essential genes occupying the first position of operons, and the lower the proportion of non-essential genes occupying the last position of operons. Conversely, the lower the proportion of essential genes in the genes in operons, the lower the proportion of essential genes occupying the first position of operons, and the higher the proportion of non-essential genes occupying the last position of operons.

#### Position preference of essential genes in general positions of operons

**Fig 2:**
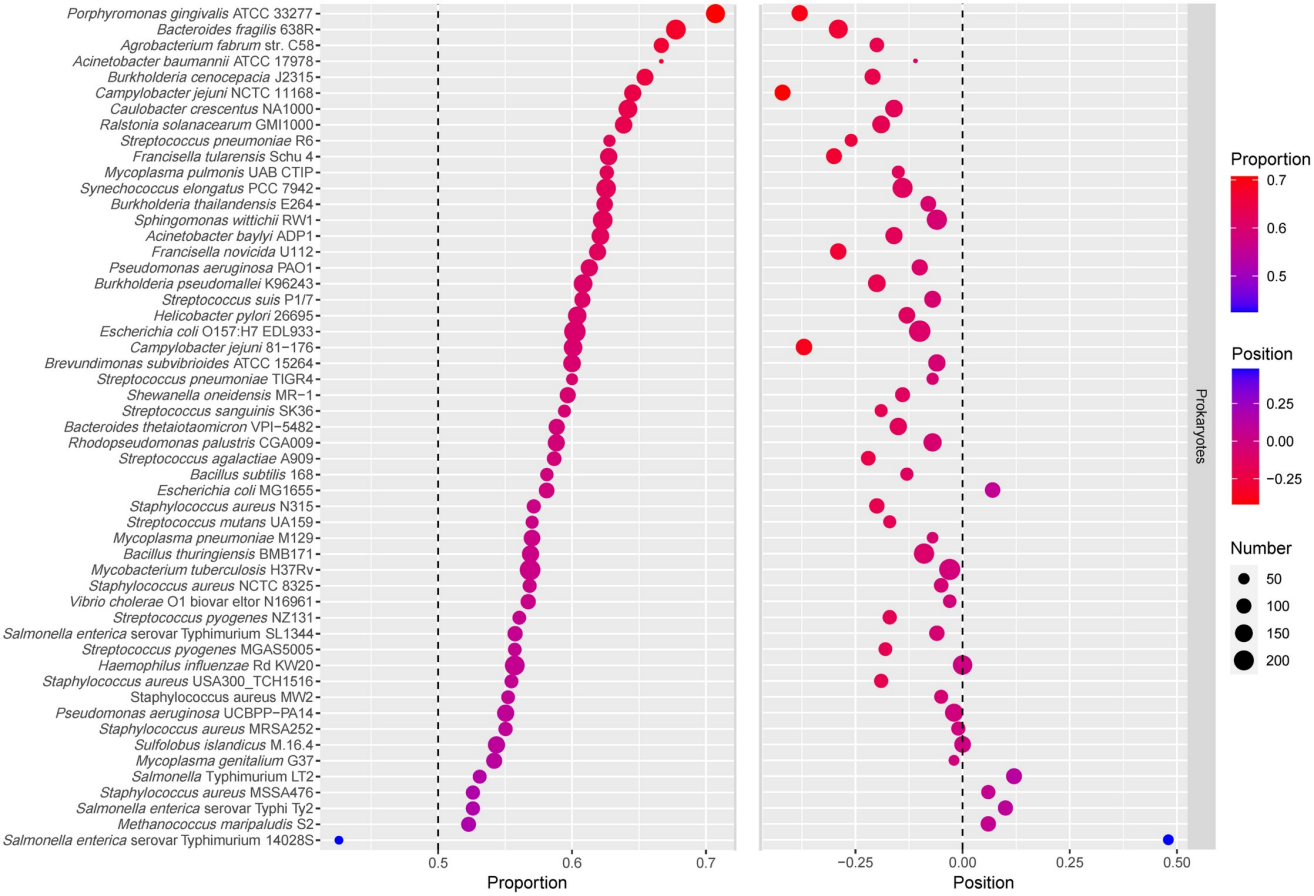
Bubblechart of essential genes proportion and the relative positions. In the left part of the figure, the size of the dot represents the number of essential genes occupying the first half of operons, and the color of the dot represents the proportion of essential genes occupying the first half of operons. The part on the left is sorted according to the proportion of essential genes in the first half of operons from high to low. In the right part of the figure, the size of the dot represents the number of operons, and the color of the dot represents the relative positions of essential genes.

It should be noted that if all the genes in an operon are essential genes, the first position is occupied by an essential gene. Therefore, operons whose genes are exclusively essential genes were removed from analysis, and then the distribution of essential genes in hybrid operons (operons containing both essential and non-essential genes), was analyzed again (S2 Table in S1 File). It was found that among 53 prokaryotic genomes, the number of genomes in which essential genes occupy the first position in more than 50% of the operons was reduced from 44 to 19 under this analysis (S2 Table in S1 File). The average position of essential genes in hybrid operons and the proportion of essential genes in the first half of the hybrid operons were studied (Table 1). Consequently, by analyzing the average positions of essential and non-essential genes in hybrid operons of 53 prokaryotic genomes, it was found that essential genes preferentially occupied the front positions of operons compared to non-essential genes (*P* = 0.004257, Student’s *t*-test). We also calculated the D_EG_, the relative position of the essential genes in operons, which is defined in Eq (4). If the relative position D_EG_ is negative, it means that the average position of essential genes is in front of the average position of all genes, whereas if the relative position D_EG_ is positive, it means that the average position of essential genes is behind the average position of all genes. As shown in Fig 2, the relative positions of essential genes in most genomes were negative, indicating that essential genes were biased toward the front positions of operons. Compared with the random arrangement result, the relative position of essential genes is different from zero, and essential genes tend to be located in the front positions of operons (*P* = 9.772e-07, Student’s *t*-test).

**Table 1:** The average position distribution of essential and non-essential genes in operons and the proportion of essential genes in the first half of operons.

Organism	Condition	RefSeq	${\bar{X}}_{EG}$	${\bar{X}}_{NEG}$	$\bar{X}$	D_EG_	No. EG in the first half of operons	No. EG in operons	Proportion	No. Operons
*Bacillus subtilis* 168	Rich	NC_000964	2.18	2.33	2.31	-0.13	68	117	58.12%	72
*Staphylococcus aureus* N315	Rich	NC_002745	2.24	2.41	2.44	-0.20	80	140	57.14%	105
*Haemophilus influenzae* Rd KW20	Rich	NC_000907	2.30	2.30	2.30	0.00	185	332	55.72%	189
*Mycoplasma genitalium* G37	Rich	NC_000908	3.52	4.15	3.55	-0.03	110	203	54.19%	44
*Streptococcus pneumoniae* TIGR4	Rich	NC_003028	2.44	2.59	2.51	-0.07	51	85	60.00%	60
*Streptococcus pneumoniae* R6	Rich	NC_003098	2.19	2.52	2.45	-0.26	54	86	62.79%	68
*Helicobacter pylori* 26695	Rich	NC_000915	2.90	3.07	3.02	-0.12	157	260	60.38%	129
*Mycobacterium tuberculosis* H37Rv	Rich	NC_000962	2.25	2.33	2.28	-0.03	207	364	56.87%	229
*Salmonella* Typhimurium LT2	Rich	NC_003197	2.44	2.27	2.32	0.12	77	145	53.10%	116
*Francisella novicida* U112	Rich	NC_008601	2.23	2.72	2.52	-0.29	130	210	61.90%	125
*Acinetobacter baylyi* ADP1	Rich	NC_005966	2.01	2.34	2.17	-0.16	141	227	62.11%	140
*Mycoplasma pulmonis* UAB CTIP	Rich	NC_002771	2.17	2.58	2.31	-0.14	82	131	62.60%	70
*Pseudomonas aeruginosa* UCBPP-PA14	Rich	NC_008463	2.53	2.54	2.54	-0.01	131	238	55.04%	156
*Staphylococcus aureus* NCTC 8325	Rich	NC_007795	2.12	2.15	2.17	-0.05	79	139	56.83%	93
*Escherichia coli* MG1655	Rich	NC_000913	2.41	2.41	2.34	0.07	104	179	58.10%	108
*Caulobacter crescentus* NA1000	Rich	NC_011916	2.07	2.49	2.23	-0.16	163	254	64.17%	149
*Streptococcus sanguinis* SK36	Rich	NC_009009	2.10	2.44	2.29	-0.19	63	106	59.43%	70
*Porphyromonas gingivalis* ATCC 33277	Rich	NC_010729	1.96	2.78	2.34	-0.38	169	239	70.71%	121
*Bacteroides thetaiotaomicron* VPI-5482	Rich	NC_004663	2.23	2.39	2.38	-0.15	113	192	58.85%	143
*Burkholderia thailandensis* E264	Rich.	NC_007651	2.15	2.33	2.23	-0.08	118	189	62.43%	113
*Salmonella enterica* serovar Typhimurium 14028S	Rich	NC_016856	2.90	2.34	2.42	0.48	23	54	42.59%	44
*Sphingomonas wittichii* RW1	Rich	NC_009511	2.16	2.33	2.22	-0.06	185	297	62.29%	208
*Shewanella oneidensis* MR-1	Rich	NC_004347	2.29	2.67	2.43	-0.14	111	186	59.68%	100
*Campylobacter jejuni* NCTC 11168	Rich	NC_002163	2.95	3.55	3.38	-0.43	131	203	64.53%	117
*Salmonella enterica* serovar SL1344	Rich	NC_016810	2.20	2.30	2.26	-0.06	97	174	55.75%	106
*Salmonella enterica* serovar Typhi Ty2	Rich	NC_004631	2.30	2.19	2.21	0.09	81	154	52.60%	104
*Bacteroides fragilis* 638R	Rich.	NC_016776	2.00	2.54	2.29	-0.29	187	276	67.75%	176
*Burkholderia pseudomallei* K96243	Rich	NC_006350	2.36	2.69	2.55	-0.19	163	268	60.82%	150
*Pseudomonas aeruginosa* PAO1	Rich	NC_002516	2.40	2.59	2.50	-0.10	133	217	61.29%	121
*Streptococcus pyogenes* MGAS5005	Todd-Hewitt	NC_007297	2.26	2.45	2.44	-0.18	73	131	55.73%	81
*Streptococcus pyogenes* NZ131	Todd-Hewitt	NC_011375	2.09	2.24	2.26	-0.17	74	132	56.06%	88
*Synechococcus elongatus* PCC 7942	Rich	NC_007604	1.85	2.12	1.99	-0.14	182	291	62.54%	205
*Rhodopseudomonas palustris* CGA009	Rich	NC_005296	1.93	2.02	2.00	-0.07	130	221	58.82%	162
*Streptococcus agalactiae* A909	Rich	NC_007432	2.09	2.38	2.32	-0.23	88	150	58.67%	95
*Acinetobacter baumannii* ATCC 17978	Murine model of pneumonia	NC_009085	1.61	1.86	1.71	-0.10	10	15	66.67%	14
*Agrobacterium fabrum* str. C58	Rich	NC_003062	1.87	2.26	2.06	-0.19	96	144	66.67%	93
*Brevundimonas subvibrioides* ATCC 15264	Rich	NC_014375	2.28	2.52	2.33	-0.05	141	235	60.00%	142
*Bacillus thuringiensis* BMB171	Rich	NC_014171	2.13	2.22	2.22	-0.09	132	232	56.90%	207
*Campylobacter jejuni* 81–176	Rich	NC_008787	2.78	3.26	3.15	-0.37	161	268	60.07%	127
*Francisella tularensis* Schu 4	Rich	NC_006570	2.23	2.78	2.53	-0.30	133	212	62.74%	115
*Streptococcus mutans* UA159	Rich	NC_004350	2.31	2.49	2.49	-0.18	65	114	57.02%	70
*Escherichia coli* O157:H7 EDL933	Rich	NC_002655	2.23	2.43	2.33	-0.10	227	377	60.21%	239
*Ralstonia solanacearum* GMI1000	Rich	NC_003295	2.18	2.47	2.36	-0.18	136	213	63.85%	151
*Streptococcus suis* P1/7	Columbia blood base agar	NC_012925	2.06	2.17	2.13	-0.07	110	181	60.77%	131
*Staphylococcus aureus* USA300_TCH1516	Rich	NC_010079	2.22	2.41	2.41	-0.19	76	137	55.47%	87
*Staphylococcus aureus* MW2	Rich	NC_003923	2.30	2.36	2.36	-0.06	74	134	55.22%	84
*Staphylococcus aureus* MSSA476	Rich	NC_002953	2.50	2.43	2.43	0.07	81	154	52.60%	88
*Staphylococcus aureus* MRSA252	Rich	NC_002952	2.44	2.53	2.45	-0.01	82	149	55.03%	87
*Burkholderia cenocepacia* J2315	Rich	NC_011000	2.01	2.47	2.22	-0.21	125	191	65.45%	118
*Vibrio cholerae* O1 biovar eltor N16961	Rich	NC_002505	2.88	3.00	2.90	-0.02	97	171	56.73%	77
*Mycoplasma pneumoniae* M129	Rich	NC_000912	3.30	3.85	3.37	-0.07	122	214	57.01%	53
*Methanococcus maripaludis* S2	Rich	NC_005791	2.21	2.16	2.15	0.06	92	176	52.27%	106
*Sulfolobus islandicus* M.16.4	Rich	NC_012726	2.46	2.48	2.46	0.00	131	241	54.36%	130

We also studied the proportion of essential genes in the first half of hybrid operons. Please note that if the number of genes in the operon is odd, the middle gene is considered to be in the first half of the operon. The bubblechart of the relative position of essential genes in operons and the proportion of essential genes occupying the first half of operons is shown in Fig 2. It was found that the relative positions of essential genes in the genomes with a lower proportion of essential genes occupying the first half of operons tended to be positive. The Pearson correlation coefficient between them was −0.78. By analyzing the relative position of essential genes in operons and the proportion of essential genes occupying the first half of operons in 53 prokaryotic genomes, it was confirmed that essential genes tend to occupy the front positions of operons. Moreover, the Pearson correlation coefficients between D_EG_ and the proportion of essential genes in operons was only 0.02, while the Pearson correlation coefficients between the proportion of essential genes occupying the first half of operons and the proportion of essential genes in operons was −0.12. This indicates that these results are independent of the proportion of essential genes in operons. Therefore, compared to the previous result that essential genes tend to occupy the first position of operons [35], the present conclusion on the position preference of essential genes in operons is more general and reliable.

### The possible reason for position preference of essential genes in operons

Depending on whether the operon contains essential genes, operons can be divided into three categories: operons containing only essential genes, operons containing both essential and non-essential genes, and operons containing only non-essential genes. By analyzing these three types of operons in 53 prokaryotic genomes, we found that essential genes have an impact on both gene number and the location of operons. Operons containing essential genes were more biased to be on the leading strand, and the average gene number of operons containing essential and non-essential genes was higher (S4 Table in S1 File).

Previous studies have shown that there is a strong relationship between gene expression and the number, length, and order of genes in operons [41]. In operons, the distance from the start of the gene to the end of the operon is defined as the transcription distance. Gene expression increases with an increase in the transcription distance; that is, gene expression increases with an increase in the length of the operon [42, 43]. Changes in the order of genes in operons also affect gene expression. The gene farthest from the end of the operon (or the gene closer to the promoter) was always more expressed. That is, the expression level of the gene in the first position is higher than that of the same gene at other positions [41]. In 46 prokaryotic genomes, the average position of essential genes is generally in front of the average position of non-essential genes, which indicates that essential genes tend to have a higher expression level than non-essential genes (Table 1). Operons containing essential and non-essential genes have more genes, thereby increasing the expression of genes in operons. This is consistent with the fact that essential genes are crucial genes with higher expression levels and encode proteins that perform important functions. It also explains the fact that essential genes tend to be located in operons rather than alone. This work will be of great significance for understanding the functional basis of genome organization and the practical application of synthetic biology.

## Conclusion

In the present study, the position preference of essential genes in prokaryotic operons was explored systematically. The result of a previous study showed that essential genes tend to occupy the first position of operons was related to the proportion of essential genes in operons. To solve this problem, a new index, the average position of genes in an operon, is proposed, which better reflects the position preference of essential genes in operons. Thus, previous shortcomings were avoided, and more general and reliable conclusions were reached. Our work provides new insights into related research on synthetic biology, such as the construction of cell factories and the design of artificial genomes.

## Supporting information

S1 File (DOCX)

## Abbreviations

COG: cluster of orthologous group
EG: essential gene
NEG: non-essential gene
