# Supplementary material for: Position preference of essential genes in prokaryotic operons
Source: PLoS One. 2021 Apr 22;16(4):e0250380. doi: 10.1371/journal.pone.0250380 (PMC8061932; doi:10.1371/journal.pone.0250380)
Supplement: S1 File — (DOCX) [file pone.0250380.s001.docx]

**S1 Table. The distribution of essential genes and non-essential genes.**

| **Organism** | **RefSeq** | **EG** | | | **% EG in operon** | **NEG** | | | **% NEG in operon** | **Fisher**  **p-value** | **Odds**  **ratio** |
| --- | --- | --- | --- | --- | --- | --- | --- | --- | --- | --- | --- |
|  |  | **Operon gene** | **Non-operon gene** | **Total** |  | **Operon gene** | **Non-operon gene** | **Total** |  |  |  |
| *Bacillus subtilis* 168 | NC_000964 | 206 | 65 | 271 | 76.01% | 2298 | 1607 | 3905 | 58.85% | 1.26E-08 | 2.215842 |
| *Staphylococcus aureus* N315 | NC_002745 | 213 | 89 | 302 | 70.53% | 1291 | 990 | 2281 | 56.60% | 3.00E-06 | 1.834846 |
| *Haemophilus influenzae* Rd KW20 | NC_000907 | 441 | 201 | 642 | 68.69% | 712 | 303 | 1015 | 70.15% | 0.5468 | 0.933735 |
| *Mycoplasma genitalium* G37 | NC_000908 | 342 | 36 | 378 | 90.48% | 76 | 21 | 97 | 78.35% | 0.002433 | 2.618506 |
| *Streptococcus pneumoniae* TIGR4 | NC_003028 | 95 | 16 | 111 | 85.59% | 1315 | 679 | 1994 | 65.95% | 6.55E-06 | 3.06451 |
| *Streptococcus pneumoniae* R6 | NC_003098 | 96 | 37 | 133 | 72.18% | 1301 | 608 | 1909 | 68.15% | 0.3853 | 1.212398 |
| *Helicobacter pylori* 26695 | NC_000915 | 279 | 43 | 322 | 86.65% | 910 | 341 | 1251 | 72.74% | 8.74E-08 | 2.430161 |
| *Mycobacterium tuberculosis* H37Rv | NC_000962 | 487 | 127 | 614 | 79.32% | 2076 | 1313 | 3389 | 61.26% | < 2.2E-16 | 2.424734 |
| *Salmonella* Typhimurium LT2 | NC_003197 | 153 | 77 | 230 | 66.52% | 2552 | 1759 | 4311 | 59.20% | 0.02745 | 1.369482 |
| *Francisella novicida* U112 | NC_008601 | 321 | 69 | 390 | 82.31% | 930 | 399 | 1329 | 69.98% | 8.12E-07 | 1.995186 |
| *Acinetobacter baylyi* ADP1 | NC_005966 | 348 | 151 | 499 | 69.74% | 1601 | 1207 | 2808 | 57.02% | 8.72E-08 | 1.737187 |
| *Mycoplasma pulmonis* UAB CTIP | NC_002771 | 247 | 63 | 310 | 79.68% | 299 | 173 | 472 | 63.35% | 1.06E-06 | 2.266154 |
| *Pseudomonas aeruginosa* UCBPP-PA14 | NC_008463 | 257 | 78 | 335 | 76.72% | 3403 | 2154 | 5557 | 61.24% | 5.34E-09 | 2.085312 |
| *Staphylococcus aureus* NCTC 8325 | NC_007795 | 250 | 101 | 351 | 71.23% | 1450 | 1090 | 2540 | 57.09% | 3.21E-07 | 1.860317 |
| *Escherichia coli* MG1655 | NC_000913 | 232 | 64 | 296 | 78.38% | 2388 | 1462 | 3850 | 62.03% | 6.76E-09 | 2.218932 |
| *Caulobacter crescentus* NA1000 | NC_011916 | 338 | 142 | 480 | 70.42% | 2090 | 1308 | 3398 | 61.51% | 0.000153 | 1.48952 |
| *Streptococcus sanguinis* SK36 | NC_009009 | 159 | 59 | 218 | 72.94% | 1346 | 706 | 2052 | 65.59% | 0.02901 | 1.413323 |
| *Porphyromonas gingivalis* ATCC 33277 | NC_010729 | 385 | 78 | 463 | 83.15% | 980 | 647 | 1627 | 60.23% | < 2.2E-16 | 3.257073 |
| *Bacteroides thetaiotaomicron* VPI-5482 | NC_004663 | 230 | 95 | 325 | 70.77% | 2867 | 1586 | 4453 | 64.38% | 0.0221 | 1.339215 |
| *Burkholderia thailandensis* E264 | NC_007650 | 33 | 9 | 42 | 78.57% | 1405 | 909 | 2314 | 60.72% | 0.0242 | 2.371471 |
| *Salmonella enterica* serovar Typhimurium 14028S | NC_016856 | 67 | 38 | 105 | 63.81% | 3213 | 2109 | 5322 | 60.37% | 0.5456 | 1.15726 |
| *Sphingomonas wittichii* RW1 | NC_009511 | 387 | 148 | 535 | 72.34% | 2837 | 1478 | 4315 | 65.75% | 0.002211 | 1.36219 |
| *Shewanella oneidensis* MR-1 | NC_004347 | 312 | 90 | 402 | 77.61% | 1835 | 2081 | 3916 | 46.86% | < 2.2E-16 | 3.930247 |
| *Campylobacter jejuni* NCTC 11168 | NC_002163 | 211 | 17 | 228 | 92.54% | 1180 | 271 | 1451 | 81.32% | 7.75E-06 | 2.848931 |
| *Salmonella enterica* serovar Typhimurium SL1344 | NC_016810 | 239 | 114 | 353 | 67.71% | 2471 | 1730 | 4201 | 58.82% | 0.001045 | 1.467677 |
| *Salmonella enterica* serovar Typhi Ty2 | NC_004631 | 264 | 94 | 358 | 73.74% | 2320 | 1635 | 3955 | 58.66% | 1.46E-08 | 1.97896 |
| *Bacteroides fragilis* 638R | NC_016776 | 407 | 140 | 547 | 74.41% | 2514 | 1320 | 3834 | 65.57% | 3.63E-05 | 1.526283 |
| *Burkholderia pseudomallei* K96243 | NC_006350 | 339 | 84 | 423 | 80.14% | 1999 | 977 | 2976 | 67.17% | 3.30E-08 | 1.972074 |
| *Pseudomonas aeruginosa* PAO1 | NC_002516 | 272 | 64 | 336 | 80.95% | 3181 | 2054 | 5235 | 60.76% | 1.39E-14 | 2.743805 |
| *Streptococcus pyogenes* MGAS5005 | NC_007297 | 158 | 69 | 227 | 69.60% | 1090 | 548 | 1638 | 66.54% | 0.3677 | 1.151176 |
| *Streptococcus pyogenes* NZ131 | NC_011375 | 172 | 69 | 241 | 71.37% | 924 | 535 | 1459 | 63.33% | 0.01645 | 1.443014 |
| *Synechococcus elongatus* PCC 7942 | NC_007604 | 422 | 258 | 680 | 62.06% | 1051 | 934 | 1985 | 52.95% | 3.88E-05 | 1.453358 |
| *Rhodopseudomonas palustris* CGA009 | NC_005296 | 357 | 195 | 552 | 64.67% | 2227 | 2034 | 4261 | 52.26% | 3.64E-08 | 1.671922 |
| *Streptococcus agalactiae* A909 | NC_007432 | 238 | 77 | 315 | 75.56% | 1084 | 597 | 1681 | 64.49% | 0.000123 | 1.701853 |
| *Acinetobacter baumannii* ATCC 17978 | NC_009085 | 40 | 114 | 154 | 25.97% | 714 | 2483 | 3197 | 22.33% | 0.279 | 1.220147 |
| *Agrobacterium fabrum* str. C58 | NC_003062 | 212 | 94 | 306 | 69.28% | 1240 | 1219 | 2459 | 50.43% | 3.06E-10 | 2.21652 |
| *Brevundimonas subvibrioides* ATCC 15264 | NC_014375 | 311 | 101 | 412 | 75.49% | 1937 | 978 | 2915 | 66.45% | 0.000203 | 1.554511 |
| *Bacillus thuringiensis* BMB171 | NC_014171 | 245 | 268 | 513 | 47.76% | 2176 | 2392 | 4568 | 47.64% | 0.9629 | 1.004924 |
| *Campylobacter jejuni* 81-176 | NC_008787 | 350 | 34 | 384 | 91.15% | 1026 | 243 | 1269 | 80.85% | 7.58E-07 | 2.436974 |
| *Francisella tularensis* Schu 4 | NC_006570 | 379 | 74 | 453 | 83.66% | 699 | 500 | 1199 | 58.30% | < 2.2E-16 | 3.660903 |
| *Streptococcus mutans* UA159 | NC_004350 | 142 | 55 | 197 | 72.08% | 1118 | 645 | 1763 | 63.41% | 0.01847 | 1.489221 |
| *Escherichia coli* O157:H7 EDL933 | NC_002655 | 636 | 396 | 1032 | 61.63% | 2692 | 1574 | 4266 | 63.10% | 0.3891 | 0.939068 |
| *Ralstonia solanacearum* GMI1000 | NC_003295 | 248 | 133 | 381 | 65.09% | 1997 | 1059 | 3056 | 65.35% | 0.9545 | 0.988821 |
| *Streptococcus suis* P1/7 | NC_012925 | 243 | 114 | 357 | 68.07% | 988 | 479 | 1467 | 67.35% | 0.8501 | 1.033412 |
| *Staphylococcus aureus* USA300_TCH1516 | NC_010079 | 207 | 66 | 273 | 75.82% | 1361 | 1027 | 2388 | 56.99% | 1.09E-09 | 2.365885 |
| *Staphylococcus aureus* MW2 | NC_003923 | 194 | 62 | 256 | 75.78% | 1336 | 1112 | 2448 | 54.58% | 3.15E-11 | 2.603366 |
| *Staphylococcus aureus* MSSA476 | NC_002953 | 229 | 76 | 305 | 75.08% | 1280 | 986 | 2266 | 56.49% | 2.81E-10 | 2.320261 |
| *Staphylococcus aureus* MRSA252 | NC_002952 | 224 | 71 | 295 | 75.93% | 1324 | 1031 | 2355 | 56.22% | 3.19E-11 | 2.455851 |
| *Burkholderia cenocepacia* J2315 | NC_011000 | 262 | 78 | 340 | 77.06% | 1920 | 1204 | 3124 | 61.46% | 7.59E-09 | 2.105927 |
| *Vibrio cholerae* O1 biovar eltor N16961 | NC_002505 | 247 | 68 | 315 | 78.41% | 1452 | 974 | 2426 | 59.85% | 4.36E-11 | 2.43581 |
| *Mycoplasma pneumoniae* M129 | NC_000912 | 298 | 44 | 342 | 87.13% | 181 | 166 | 347 | 52.16% | < 2.2E-16 | 6.194102 |
| *Methanococcus maripaludis* S2 | NC_005791 | 350 | 169 | 519 | 67.44% | 716 | 487 | 1203 | 59.52% | 0.002042 | 1.408347 |
| *Sulfolobus islandicus* M.16.4 | NC_012726 | 335 | 106 | 441 | 75.96% | 1368 | 926 | 2294 | 59.63% | 3.31E-11 | 2.138703 |

**S2 Table. The distribution of essential genes in the first position and non-essential genes in the last position of operons.**

| **Organism** | **Operons^a^** | | | | |  | **Operons^b^** | | | | | |  |
| --- | --- | --- | --- | --- | --- | --- | --- | --- | --- | --- | --- | --- | --- |
|  | **No. operons^c^** | **No. operons^d^** | **Fisher p-value** | **odds ratio** | **No. EGs in operons^a^** |  | **No.**  **Operons^e^** | **No.**  **Operons^f^** | **No. operons^g^** | **Fisher**  **p-value** | **odds ratio** | **No. EGs in operons^b^** | **No.**  **operons** |
| *NNBacillus subtilis* 168 | 58(58.00%) | 42 | 0.03364 | 1.900793 | 206(58.86%) |  | 30(41.67%) | 39(54.17%) | 33 | 0.4048 | 0.717662 | 117(44.83%) | 818 |
| *Staphylococcus aureus* N315 | 62(48.82%) | 65 | 0.8019 | 0.910162 | 213(44.28%) |  | 40(38.10%) | 63(60.00%) | 42 | 0.005648 | 0.446211 | 140(34.31%) | 503 |
| *Haemophilus influenzae* Rd KW20 | 137(58.55%) | 97 | 0.0003023 | 1.991803 | 441(55.96%) |  | 92(48.68%) | 99(52.38%) | 90 | 0.4106 | 0.826869 | 332(48.9%) | 374 |
| *Mycoplasma genitalium* G37 | 69(82.14%) | 15 | < 2.2E-16 | 20.610990 | 342(84.03%) |  | 29(65.91%) | 13(29.55%) | 31 | 0.0002442 | 5.560814 | 203(75.75%) | 89 |
| *Streptococcus pneumoniae* TIGR4 | 29(44.62%) | 36 | 0.2926 | 0.651117 | 95(37.85%) |  | 24(40.00%) | 41(68.33%) | 19 | 0.0001079 | 0.217807 | 85(35.27%) | 466 |
| *Streptococcus pneumoniae* R6 | 37(51.39%) | 35 | 0.8677 | 1.116687 | 96(34.91%) |  | 33(48.53%) | 46(67.65%) | 22 | 6.86E-05 | 0.231462 | 86(32.45%) | 473 |
| *Helicobacter pylori* 26695 | 49(36.03%) | 87 | 6.36E-06 | 0.318639 | 279(41.64%) |  | 42(32.56%) | 87(67.44%) | 42 | 3.16E-08 | 0.234495 | 260(39.94%) | 319 |
| *Mycobacterium tuberculosis* H37Rv | 145(53.31%) | 127 | 0.1448 | 1.302898 | 487(51.81%) |  | 102(44.54%) | 134(58.52%) | 95 | 0.0003725 | 0.503385 | 364(44.55%) | 898 |
| *Salmonella* Typhimurium LT2 | 39(32.50%) | 81 | 9.05E-08 | 0.233371 | 153(35.58%) |  | 35(30.17%) | 69(59.48%) | 47 | 0.005709 | 0.465554 | 145(34.36%) | 879 |
| *Francisella novicida* U112 | 94(59.87%) | 63 | 0.000685 | 2.220436 | 321(52.11%) |  | 62(49.60%) | 84(67.20%) | 41 | 8.19E-08 | 0.239729 | 210(41.58%) | 373 |
| *Acinetobacter baylyi* ADP1 | 120(73.39%) | 64 | 7.61E-09 | 3.502461 | 348(59.18%) |  | 76(54.29%) | 82(58.57%) | 58 | 0.00588 | 0.501559 | 227(48.61%) | 643 |
| *Mycoplasma pulmonis* UAB CTIP | 80(73.39%) | 29 | 5.24E-12 | 7.528056 | 247(66.76%) |  | 41(58.57%) | 43(61.43%) | 27 | 0.01097 | 0.396987 | 131(51.57%) | 175 |
| *Pseudomonas aeruginosa* UCBPP-PA14 | 72(43.90%) | 92 | 0.03573 | 0.613389 | 257(39.18%) |  | 64(41.03%) | 93(59.62%) | 63 | 0.0009938 | 0.460073 | 238(37.36%) | 1205 |
| *Staphylococcus aureus* NCTC 8325 | 82(62.60%) | 49 | 7.11E-05 | 2.789023 | 250(59.38%) |  | 44(47.31%) | 52(55.91%) | 41 | 0.1423 | 0.623263 | 139(44.84%) | 557 |
| *Escherichia coli* MG1655 | 68(53.54%) | 59 | 0.3154 | 1.326845 | 232(51.56%) |  | 49(45.37%) | 62(57.41%) | 46 | 0.04098 | 0.552008 | 179(45.09%) | 853 |
| *Caulobacter crescentus* NA1000 | 123(67.21%) | 60 | 5.96E-11 | 4.184601 | 338(56.24%) |  | 89(59.73%) | 100(67.11%) | 49 | 4.98E-09 | 0.241353 | 254(49.13%) | 844 |
| *Streptococcus sanguinis* SK36 | 50(57.47%) | 37 | 0.06854 | 1.819767 | 159(52.3%) |  | 33(47.14%) | 46(65.71%) | 24 | 0.0003504 | 0.274919 | 106(42.23%) | 489 |
| *Porphyromonas gingivalis* ATCC 33277 | 144(83.72%) | 28 | < 2.2E-16 | 26.059730 | 385(65.14%) |  | 93(76.86%) | 93(76.86%) | 28 | < 2.2E-16 | 0.091757 | 239(53.71%) | 455 |
| *Bacteroides thetaiotaomicron* VPI-5482 | 75(46.88%) | 85 | 0.3143 | 0.779167 | 230(39.93%) |  | 58(40.56%) | 88(61.54%) | 55 | 0.0001451 | 0.391983 | 192(35.69%) | 1016 |
| *Burkholderia thailandensis* E264 | 92(62.59%) | 55 | 2.45E-05 | 2.787809 | 277(57.83%) |  | 58(51.33%) | 69(61.06%) | 44 | 0.001356 | 0.408315 | 189(48.34%) | 443 |
| *Salmonella enterica* serovar Typhimurium 14028S | 16(33.33%) | 32 | 0.002037 | 0.253937 | 67(36.81%) |  | 12(27.27%) | 23(52.27%) | 21 | 0.8313 | 0.835381 | 54(31.95%) | 1066 |
| *Sphingomonas wittichii* RW1 | 140(58.33%) | 100 | 0.0003604 | 1.957214 | 387(48.13%) |  | 108(51.92%) | 139(66.83%) | 69 | 8.54E-12 | 0.247317 | 297(41.6%) | 1095 |
| *Shewanella oneidensis* MR-1 | 102(70.34%) | 43 | 4.98E-12 | 5.589118 | 312(60.94%) |  | 57(57.00%) | 64(64.00%) | 36 | 0.0001228 | 0.318341 | 186(48.19%) | 760 |
| *Campylobacter jejuni* NCTC 11168 | 52(43.33%) | 68 | 0.05258 | 0.586089 | 211(30.98%) |  | 49(41.88%) | 94(80.34%) | 23 | < 2.2E-16 | 0.060854 | 203(30.16%) | 347 |
| *Salmonella enterica* serovar Typhimurium SL1344 | 76(58.46%) | 54 | 0.009065 | 1.975505 | 239(54.57%) |  | 52(49.06%) | 62(58.49%) | 44 | 0.01932 | 0.505304 | 174(46.65%) | 881 |
| *Salmonella enterica* serovar Typhi Ty2 | 76(54.68%) | 63 | 0.1499 | 1.453270 | 264(56.77%) |  | 41(39.42%) | 56(53.85%) | 48 | 0.3317 | 0.735801 | 154(43.38%) | 839 |
| *Bacteroides fragilis* 638R | 155(69.20%) | 69 | 4.23E-16 | 5.025966 | 407(53.48%) |  | 107(60.80%) | 126(71.59%) | 50 | 4.56E-16 | 0.158423 | 276(43.81%) | 956 |
| *Burkholderia pseudomallei* K96243 | 105(59.66%) | 71 | 0.0004197 | 2.182088 | 339(49.34%) |  | 79(52.67%) | 102(68.00%) | 48 | 6.22E-10 | 0.222678 | 268(43.51%) | 741 |
| *Pseudomonas aeruginosa* PAO1 | 75(52.45%) | 68 | 0.478 | 1.215636 | 272(50.56%) |  | 53(43.80%) | 72(59.50%) | 49 | 0.004578 | 0.464667 | 217(44.93%) | 1139 |
| *Streptococcus pyogenes* MGAS5005 | 51(54.26%) | 43 | 0.3072 | 1.404114 | 158(46.2%) |  | 38(46.91%) | 45(55.56%) | 36 | 0.2086 | 0.641798 | 131(41.59%) | 403 |
| *Streptococcus pyogenes* NZ131 | 56(52.83%) | 50 | 0.4923 | 1.253045 | 172(49.28%) |  | 38(43.18%) | 47(53.41%) | 41 | 0.4511 | 0.762174 | 132(42.72%) | 372 |
| *Synechococcus elongatus* PCC 7942 | 163(63.67%) | 93 | 8.48E-10 | 3.064790 | 422(56.95%) |  | 112(54.63%) | 127(61.95%) | 78 | 1.92E-06 | 0.378144 | 291(47.7%) | 548 |
| *Rhodopseudomonas palustris* CGA009 | 131(62.98%) | 77 | 1.74E-07 | 2.886672 | 357(57.4%) |  | 85(52.47%) | 101(62.35%) | 61 | 1.34E-05 | 0.365982 | 221(45.47%) | 900 |
| *Streptococcus agalactiae* A909 | 80(65.04%) | 43 | 3.79E-06 | 3.442476 | 238(54.97%) |  | 52(54.74%) | 60(63.16%) | 35 | 0.0004659 | 0.342311 | 150(43.48%) | 437 |
| *Acinetobacter baumannii* ATCC 17978 | 20(76.92%) | 6 | 0.0002321 | 10.479000 | 40(67.8%) |  | 8(57.14%) | 9(64.29%) | 5 | 0.2568 | 0.322675 | 15(44.12%) | 336 |
| *Agrobacterium fabrum* str. C58 | 82(71.30%) | 33 | 1.31E-10 | 6.118824 | 212(59.05%) |  | 60(64.52%) | 62(66.67%) | 31 | 9.12E-06 | 0.252024 | 144(49.48%) | 533 |
| *Brevundimonas subvibrioides* ATCC 15264 | 103(60.23%) | 68 | 0.0002257 | 2.288610 | 311(52.09%) |  | 74(52.11%) | 91(64.08%) | 51 | 3.21E-06 | 0.315457 | 235(45.11%) | 731 |
| *Bacillus thuringiensis* BMB171 | 91(42.72%) | 122 | 0.0036 | 0.557139 | 245(33.75%) |  | 85(41.06%) | 122(58.94%) | 85 | 0.0003898 | 0.486286 | 232(32.54%) | 877 |
| *Campylobacter jejuni* 81-176 | 87(56.86%) | 66 | 0.02206 | 1.734457 | 350(46.3%) |  | 61(48.03%) | 90(70.87%) | 37 | 3.58E-11 | 0.170363 | 268(39.76%) | 354 |
| *Francisella tularensis* Schu 4 | 112(71.34%) | 45 | 3.90E-14 | 6.153534 | 379(59.87%) |  | 70(60.87%) | 77(66.96%) | 38 | 4.26E-07 | 0.245177 | 212(45.49%) | 326 |
| *Streptococcus mutans* UA159 | 44(53.66%) | 38 | 0.435 | 1.338296 | 142(46.41%) |  | 32(45.71%) | 40(57.14%) | 30 | 0.1279 | 0.564841 | 114(41.01%) | 420 |
| *Escherichia coli* O157:H7 EDL933 | 207(62.92%) | 122 | 4.55E-11 | 2.874015 | 636(56.04%) |  | 117(48.95%) | 138(57.74%) | 101 | 0.0009707 | 0.536360 | 377(43.04%) | 1048 |
| *Ralstonia solanacearum* GMI1000 | 87(53.05%) | 77 | 0.3203 | 1.275640 | 248(41.47%) |  | 74(49.01%) | 97(64.24%) | 54 | 1.15E-06 | 0.311201 | 213(37.83%) | 722 |
| *Streptococcus suis* P1/7 | 90(57.69%) | 66 | 0.0091 | 1.855770 | 243(49.69%) |  | 65(49.62%) | 82(62.60%) | 49 | 7.11E-05 | 0.358549 | 181(42.39%) | 418 |
| *Staphylococcus aureus* USA300_TCH1516 | 72(61.02%) | 46 | 0.001091 | 2.440308 | 207(51.49%) |  | 41(47.13%) | 53(60.92%) | 34 | 0.006188 | 0.413710 | 137(41.27%) | 516 |
| *Staphylococcus aureus* MW2 | 66(59.46%) | 45 | 0.007125 | 2.143524 | 194(52.15%) |  | 39(46.43%) | 48(57.14%) | 36 | 0.08934 | 0.564449 | 134(42.95%) | 499 |
| *Staphylococcus aureus* MSSA476 | 69(57.02%) | 52 | 0.03946 | 1.756585 | 229(55.18%) |  | 36(40.91%) | 47(53.41%) | 41 | 0.4511 | 0.762174 | 154(45.29%) | 490 |
| *Staphylococcus aureus* MRSA252 | 71(59.17%) | 49 | 0.006586 | 2.092920 | 224(53.98%) |  | 38(43.68%) | 47(54.02%) | 40 | 0.363 | 0.725673 | 149(43.82%) | 511 |
| *Burkholderia cenocepacia* J2315 | 95(66.43%) | 48 | 4.09E-08 | 3.896966 | 262(54.93%) |  | 70(59.32%) | 83(70.34%) | 35 | 5.56E-10 | 0.179295 | 191(47.04%) | 541 |
| *Vibrio cholerae* O1 biovar eltor N16961 | 60(57.69%) | 44 | 0.03726 | 1.853897 | 247(55.38%) |  | 33(42.86%) | 41(53.25%) | 36 | 0.5193 | 0.772286 | 171(46.22%) | 115 |
| *Mycoplasma pneumoniae* M129 | 61(76.25%) | 19 | 3.22E-11 | 10.119630 | 298(76.8%) |  | 34(64.15%) | 23(43.40%) | 30 | 0.2437 | 1.692746 | 214(70.39%) | 748 |
| *Methanococcus maripaludis* S2 | 101(61.96%) | 62 | 2.36E-05 | 2.645486 | 350(66.92%) |  | 44(41.51%) | 54(50.94%) | 52 | 0.8908 | 0.927629 | 176(50.43%) | 362 |
| *Sulfolobus islandicus* M.16.4 | 94(57.67%) | 69 | 0.007754 | 1.852359 | 335(55.46%) |  | 61(46.92%) | 70(53.85%) | 60 | 0.2643 | 0.735582 | 241(47.25%) | 578 |

^a^ Operons with at least one essential gene.

^b^ Operons with at least one essential gene and one non-essential gene.

^c^ Operons in which the essential genes occupy the first positions with at least one essential gene.

^d^ Operons in which non-essential genes occupy the first positions with at least one essential gene.

^e^ Operons in which the essential genes occupy the first positions with at least one essential gene and one non-essential gene.

^f^ Operons in which non-essential genes occupy the last positions with at least one essential gene and one non-essential gene.

^g^ Operons in which the essential genes occupy the last positions with at least one essential gene and one non-essential gene.

**S3 Table. Position of essential gene in operons containing two and three genes.**

| **Organism** | **Position in operons of size 2** | | | | | | | |  | **Position in operon of size 3** | | | | | | | |
| --- | --- | --- | --- | --- | --- | --- | --- | --- | --- | --- | --- | --- | --- | --- | --- | --- | --- |
|  | **1** | **2** | **Expected** | **Total** | | **X-squared** | **DF** | **P-value** |  | **1** | **2** | **3** | **Expected** | **Total** | **X-squared** | **DF** | **P-value** |
| *Bacillus subtilis* 168 | 29 | 16 | 22.5 | 45 | 3.7556 | | 1 | 0.05263 |  | 12 | 4 | 6 | 7.33 | 22 | 4.7285 | 2 | 0.09402 |
| *Staphylococcus aureus* N315 | 32 | 13 | 22.5 | 45 | 8.0222 | | 1 | 0.004621 |  | 10 | 9 | 8 | 9 | 27 | 0.22281 | 2 | 0.8946 |
| *Haemophilus influenzae* Rd KW20 | 70 | 33 | 51.5 | 103 | 13.291 | | 1 | 0.000267 |  | 21 | 20 | 11 | 17.33 | 52 | 3.5035 | 2 | 0.1735 |
| *Mycoplasma genitalium* G37 | 19 | 5 | 12 | 24 | 8.1667 | | 1 | 0.004267 |  | 18 | 2 | 0 | 6.67 | 20 | 29.205 | 2 | 4.55E-07 |
| *Streptococcus pneumoniae* TIGR4 | 12 | 6 | 9 | 18 | 2 | | 1 | 0.1573 |  | 10 | 7 | 2 | 6.33 | 19 | 5.1601 | 2 | 0.07577 |
| *Streptococcus pneumoniae* R6 | 18 | 7 | 12.5 | 25 | 4.84 | | 1 | 0.02781 |  | 6 | 4 | 5 | 5 | 15 | 0.40004 | 2 | 0.8187 |
| *Helicobacter pylori* 26695 | 15 | 18 | 16.5 | 33 | 0.27273 | | 1 | 0.6015 |  | 10 | 12 | 2 | 8 | 24 | 7.003 | 2 | 0.03015 |
| *Mycobacterium tuberculosis* H37Rv | 64 | 44 | 54 | 108 | 3.7037 | | 1 | 0.05429 |  | 31 | 19 | 13 | 21 | 63 | 8.0047 | 2 | 0.01827 |
| *Salmonella* Typhimurium LT2 | 25 | 26 | 25.5 | 51 | 0.019608 | | 1 | 0.8886 |  | 9 | 9 | 2 | 6.67 | 20 | 4.9023 | 2 | 0.08619 |
| *Francisella novicida* U112 | 37 | 19 | 28 | 56 | 5.7857 | | 1 | 0.01616 |  | 23 | 6 | 4 | 11 | 33 | 19.823 | 2 | 4.96E-05 |
| *Acinetobacter baylyi* ADP1 | 62 | 27 | 44.5 | 89 | 13.764 | | 1 | 0.000207 |  | 33 | 11 | 5 | 16.33 | 49 | 26.619 | 2 | 1.66E-06 |
| *Mycoplasma pulmonis* UAB CTIP | 35 | 10 | 22.5 | 45 | 13.889 | | 1 | 0.000194 |  | 22 | 5 | 3 | 10 | 30 | 21.805 | 2 | 1.84E-05 |
| *Pseudomonas aeruginosa* UCBPP-PA14 | 36 | 29 | 32.5 | 65 | 0.75385 | | 1 | 0.3853 |  | 14 | 8 | 6 | 9.33 | 28 | 3.7163 | 2 | 0.156 |
| *Staphylococcus aureus* NCTC 8325 | 46 | 12 | 29 | 58 | 19.931 | | 1 | 8.03E-06 |  | 19 | 8 | 6 | 11 | 33 | 8.9123 | 2 | 0.01161 |
| *Escherichia coli* MG1655 | 38 | 17 | 27.5 | 55 | 8.0182 | | 1 | 0.004631 |  | 8 | 11 | 2 | 7 | 21 | 6.0025 | 2 | 0.04972 |
| *Caulobacter crescentus* NA1000 | 65 | 19 | 42 | 84 | 25.19 | | 1 | 5.19E-07 |  | 24 | 12 | 9 | 15 | 45 | 8.4037 | 2 | 0.01497 |
| *Streptococcus sanguinis* SK36 | 19 | 15 | 17 | 34 | 0.47059 | | 1 | 0.4927 |  | 16 | 4 | 0 | 6.67 | 20 | 20.804 | 2 | 3.04E-05 |
| *Porphyromonas gingivalis* ATCC 33277 | 64 | 11 | 37.5 | 75 | 37.453 | | 1 | 9.36E-10 |  | 36 | 3 | 3 | 14 | 42 | 51.866 | 2 | 5.46E-12 |
| *Bacteroides thetaiotaomicron* VPI-5482 | 38 | 26 | 32 | 64 | 2.25 | | 1 | 0.1336 |  | 17 | 14 | 7 | 12.67 | 38 | 4.161 | 2 | 0.1249 |
| *Burkholderia thailandensis* E264 | 52 | 23 | 37.5 | 75 | 11.213 | | 1 | 0.000812 |  | 19 | 7 | 3 | 9.67 | 29 | 14.349 | 2 | 0.000766 |
| *Salmonella enterica* serovar Typhimurium 14028S | 11 | 8 | 9.5 | 19 | 0.47368 | | 1 | 0.4913 |  | 1 | 2 | 3 | 2 | 6 | 0.99935 | 2 | 0.6067 |
| *Sphingomonas wittichii* RW1 | 72 | 28 | 50 | 100 | 19.36 | | 1 | 1.08E-05 |  | 38 | 16 | 10 | 21.33 | 64 | 20.382 | 2 | 3.75E-05 |
| *Shewanella oneidensis* MR-1 | 49 | 15 | 32 | 64 | 18.062 | | 1 | 2.14E-05 |  | 22 | 2 | 2 | 8.67 | 26 | 30.775 | 2 | 2.08E-07 |
| *Campylobacter jejuni* NCTC 11168 | 15 | 15 | 15 | 30 | 0 | | 1 | 1 |  | 14 | 4 | 1 | 6.33 | 19 | 14.635 | 2 | 0.000664 |
| *Salmonella enterica* serovar Typhimurium SL1344 | 39 | 21 | 30 | 60 | 5.4 | | 1 | 0.02014 |  | 16 | 3 | 4 | 7.67 | 23 | 13.655 | 2 | 0.001083 |
| *Salmonella enterica* serovar Typhi Ty2 | 42 | 27 | 34.5 | 69 | 3.2609 | | 1 | 0.07095 |  | 12 | 4 | 3 | 6.33 | 19 | 7.6865 | 2 | 0.02142 |
| *Bacteroides fragilis* 638R | 74 | 19 | 46.5 | 93 | 32.527 | | 1 | 1.18E-08 |  | 43 | 11 | 12 | 22 | 66 | 30.099 | 2 | 2.91E-07 |
| *Burkholderia pseudomallei* K96243 | 51 | 22 | 36.5 | 73 | 11.521 | | 1 | 0.000688 |  | 19 | 6 | 6 | 10.33 | 31 | 10.906 | 2 | 0.004283 |
| *Pseudomonas aeruginosa* PAO1 | 37 | 21 | 29 | 58 | 4.4138 | | 1 | 0.03565 |  | 14 | 12 | 5 | 10.33 | 31 | 4.3254 | 2 | 0.115 |
| *Streptococcus pyogenes* MGAS5005 | 23 | 13 | 18 | 36 | 2.7778 | | 1 | 0.09558 |  | 11 | 7 | 4 | 7.33 | 22 | 3.3655 | 2 | 0.1859 |
| *Streptococcus pyogenes* NZ131 | 27 | 22 | 24.5 | 49 | 0.5102 | | 1 | 0.4751 |  | 12 | 8 | 3 | 7.67 | 23 | 5.3068 | 2 | 0.07041 |
| *Synechococcus elongatus* PCC 7942 | 97 | 40 | 68.5 | 137 | 23.715 | | 1 | 1.12E-06 |  | 37 | 16 | 10 | 21 | 63 | 19.15 | 2 | 6.95E-05 |
| *Rhodopseudomonas palustris* CGA009 | 79 | 26 | 52.5 | 105 | 26.752 | | 1 | 2.31E-07 |  | 28 | 16 | 11 | 18.33 | 55 | 8.3316 | 2 | 0.01552 |
| *Streptococcus agalactiae* A909 | 37 | 14 | 25.5 | 51 | 10.373 | | 1 | 0.001279 |  | 21 | 6 | 4 | 10.33 | 31 | 16.714 | 2 | 0.000235 |
| *Acinetobacter baumannii* ATCC 17978 | 16 | 3 | 9.5 | 19 | 8.8947 | | 1 | 0.00286 |  | 4 | 1 | 2 | 2.33 | 7 | 2.0004 | 2 | 0.3678 |
| *Agrobacterium fabrum* str. C58 | 43 | 12 | 27.5 | 55 | 17.473 | | 1 | 2.92E-05 |  | 22 | 7 | 2 | 10.33 | 31 | 20.973 | 2 | 2.79E-05 |
| *Brevundimonas subvibrioides* ATCC 15264 | 49 | 16 | 32.5 | 65 | 16.754 | | 1 | 4.26E-05 |  | 24 | 9 | 12 | 15 | 45 | 8.4025 | 2 | 0.01498 |
| *Bacillus thuringiensis* BMB171 | 45 | 51 | 48 | 96 | 0.375 | | 1 | 0.5403 |  | 24 | 16 | 15 | 18.33 | 55 | 2.6566 | 2 | 0.2649 |
| *Campylobacter jejuni* 81-176 | 28 | 11 | 19.5 | 39 | 7.4103 | | 1 | 0.006485 |  | 13 | 7 | 3 | 7.67 | 23 | 6.6113 | 2 | 0.03668 |
| *Francisella tularensis* Schu 4 | 36 | 15 | 25.5 | 51 | 8.6471 | | 1 | 0.003276 |  | 29 | 4 | 3 | 12 | 36 | 36.174 | 2 | 1.4E-08 |
| *Streptococcus mutans* UA159 | 19 | 10 | 14.5 | 29 | 2.7931 | | 1 | 0.09467 |  | 12 | 3 | 4 | 6.33 | 19 | 7.6861 | 2 | 0.02143 |
| *Escherichia coli* O157:H7 EDL933 | 106 | 38 | 72 | 144 | 32.111 | | 1 | 1.46E-08 |  | 33 | 11 | 18 | 20.67 | 62 | 12.229 | 2 | 0.002211 |
| *Ralstonia solanacearum* GMI1000 | 43 | 23 | 33 | 66 | 6.0606 | | 1 | 0.01382 |  | 13 | 17 | 9 | 13 | 39 | 2.4638 | 2 | 0.2917 |
| *Streptococcus suis* P1/7 | 44 | 22 | 33 | 66 | 7.3333 | | 1 | 0.006769 |  | 28 | 14 | 6 | 16 | 48 | 15.506 | 2 | 0.00043 |
| *Staphylococcus aureus* USA300_TCH1516 | 42 | 14 | 28 | 56 | 14 | | 1 | 0.000183 |  | 14 | 6 | 6 | 8.67 | 26 | 4.9249 | 2 | 0.08522 |
| *Staphylococcus aureus* MW2 | 39 | 11 | 25 | 50 | 15.68 | | 1 | 7.50E-05 |  | 15 | 5 | 7 | 9 | 27 | 6.2239 | 2 | 0.04451 |
| *Staphylococcus aureus* MSSA476 | 37 | 13 | 25 | 50 | 11.52 | | 1 | 0.000689 |  | 14 | 7 | 8 | 9.67 | 29 | 2.9667 | 2 | 0.2269 |
| *Staphylococcus aureus* MRSA252 | 37 | 13 | 25 | 50 | 11.52 | | 1 | 0.000689 |  | 15 | 4 | 9 | 9.33 | 28 | 6.5008 | 2 | 0.03876 |
| *Burkholderia cenocepacia* J2315 | 53 | 14 | 33.5 | 67 | 22.701 | | 1 | 1.89E-06 |  | 17 | 10 | 7 | 11.33 | 34 | 4.6496 | 2 | 0.0978 |
| *Vibrio cholerae* O1 biovar eltor N16961 | 23 | 10 | 16.5 | 33 | 5.1212 | | 1 | 0.02364 |  | 13 | 4 | 1 | 6 | 18 | 13.003 | 2 | 0.001501 |
| *Mycoplasma pneumoniae* M129 | 19 | 5 | 12 | 24 | 8.1667 | | 1 | 0.004267 |  | 15 | 2 | 0 | 5.67 | 17 | 23.416 | 2 | 8.23E-06 |
| *Methanococcus maripaludis* S2 | 53 | 26 | 39.5 | 79 | 9.2278 | | 1 | 0.002384 |  | 21 | 9 | 8 | 12.67 | 38 | 8.2663 | 2 | 0.01603 |
| *Sulfolobus islandicus* M.16.4 | 47 | 20 | 33.5 | 67 | 10.881 | | 1 | 0.000972 |  | 18 | 11 | 10 | 13 | 39 | 2.925 | 2 | 0.2317 |

**S4 Table.** **The influence of essential genes on gene number and the location of operons.**

| **Organism** | **RefSeq** | **Average gene number** | | |  | **Percent in leading strand** | | | |
| --- | --- | --- | --- | --- | --- | --- | --- | --- | --- |
|  |  | **Nonessential operon** | **Hybrid operon** | **Essential operon** |  | **Nonessential operon** | **Hybrid operon** | **Essential operon** |  |
| *Bacillus subtilis* 168 | NC_000964 | 3.00 | 3.63 | 3.18 |  | 76.74% | 95.83% | 96.43% |  |
| *Staphylococcus aureus* N315 | NC_002745 | 2.72 | 3.89 | 3.32 |  | 75.00% | 93.33% | 95.45% |  |
| *Haemophilus influenzae* Rd KW20 | NC_000907 | 2.61 | 3.59 | 2.42 |  | 53.57% | 57.14% | 55.56% |  |
| *Mycoplasma genitalium* G37 | NC_000908 | 2.20 | 6.09 | 3.48 |  | 60.00% | 68.18% | 82.50% |  |
| *Streptococcus pneumoniae* TIGR4 | NC_003028 | 2.89 | 4.02 | 2.00 |  | 83.54% | 91.67% | 60.00% |  |
| *Streptococcus pneumoniae* R6 | NC_003098 | 2.80 | 3.90 | 2.50 |  | 80.05% | 91.18% | 100.00% |  |
| *Helicobacter pylori* 26695 | NC_000915 | 2.84 | 5.05 | 2.71 |  | 55.19% | 58.91% | 100.00% |  |
| *Mycobacterium tuberculosis* H37Rv | NC_000962 | 2.59 | 3.57 | 2.86 |  | 53.51% | 72.05% | 76.74% |  |
| *Salmonella* Typhimurium LT2 | NC_003197 | 3.00 | 3.64 | 2.00 |  | 58.10% | 69.83% | 100.00% |  |
| *Francisella novicida* U112 | NC_008601 | 2.94 | 4.04 | 3.47 |  | 58.33% | 60.80% | 78.13% |  |
| *Acinetobacter baylyi* ADP1 | NC_005966 | 2.97 | 3.34 | 2.75 |  | 61.22% | 61.43% | 63.64% |  |
| *Mycoplasma pulmonis* UAB CTIP | NC_002771 | 2.67 | 3.63 | 2.97 |  | 59.09% | 62.86% | 64.10% |  |
| *Pseudomonas aeruginosa* UCBPP-PA14 | NC_008463 | 2.89 | 4.08 | 2.38 |  | 53.51% | 65.38% | 62.50% |  |
| *Staphylococcus aureus* NCTC 8325 | NC_007795 | 3.00 | 3.33 | 2.92 |  | 77.70% | 94.62% | 97.37% |  |
| *Escherichia coli* MG1655 | NC_000913 | 2.99 | 3.68 | 2.79 |  | 51.66% | 68.52% | 78.95% |  |
| *Caulobacter crescentus* NA1000 | NC_011916 | 2.76 | 3.47 | 2.47 |  | 52.34% | 61.07% | 67.65% |  |
| *Streptococcus sanguinis* SK36 | NC_009009 | 2.99 | 3.59 | 3.12 |  | 83.83% | 88.57% | 94.12% |  |
| *Porphyromonas gingivalis* ATCC 33277 | NC_010729 | 2.73 | 3.68 | 2.86 |  | 47.70% | 51.24% | 54.90% |  |
| *Bacteroides thetaiotaomicron* VPI-5482 | NC_004663 | 2.95 | 3.76 | 2.24 |  | 57.71% | 59.44% | 47.06% |  |
| *Burkholderia thailandensis* E264 | NC_007650 | 2.84 | 3.46 | 2.59 |  | 60.00% | 64.60% | 85.29% |  |
| *Salmonella enterica* serovar Typhimurium 14028S | NC_016856 | 3.04 | 3.84 | 3.25 |  | 57.66% | 50.00% | 75.00% |  |
| *Sphingomonas wittichii* RW1 | NC_009511 | 2.83 | 3.43 | 2.81 |  | 56.14% | 50.48% | 31.25% |  |
| *Shewanella oneidensis* MR-1 | NC_004347 | 2.66 | 3.86 | 2.80 |  | 55.61% | 64.00% | 84.44% |  |
| *Campylobacter jejuni* NCTC 11168 | NC_002163 | 3.13 | 5.75 | 2.67 |  | 59.91% | 60.68% | 100.00% |  |
| *Salmonella enterica* serovar Typhimurium SL1344 | NC_016810 | 3.03 | 3.52 | 2.71 |  | 58.06% | 67.92% | 79.17% |  |
| *Salmonella enterica* serovar Typhi Ty2 | NC_004631 | 3.03 | 3.41 | 3.14 |  | 56.57% | 72.12% | 77.14% |  |
| *Bacteroides fragilis* 638R | NC_016776 | 2.95 | 3.58 | 2.73 |  | 54.23% | 53.41% | 60.42% |  |
| *Burkholderia pseudomallei* K96243 | NC_006350 | 2.92 | 4.11 | 2.73 |  | 58.05% | 57.33% | 76.92% |  |
| *Pseudomonas aeruginosa* PAO1 | NC_002516 | 2.93 | 3.99 | 2.50 |  | 54.82% | 66.94% | 77.27% |  |
| *Streptococcus pyogenes* MGAS5005 | NC_007297 | 2.93 | 3.89 | 2.08 |  | 79.94% | 87.65% | 76.92% |  |
| *Streptococcus pyogenes* NZ131 | NC_011375 | 2.81 | 3.51 | 2.22 |  | 76.32% | 92.05% | 72.22% |  |
| *Synechococcus elongatus* PCC 7942 | NC_007604 | 2.51 | 2.98 | 2.57 |  | 54.11% | 47.80% | 52.94% |  |
| *Rhodopseudomonas palustris* CGA009 | NC_005296 | 2.84 | 3.00 | 2.96 |  | 57.08% | 67.28% | 63.04% |  |
| *Streptococcus agalactiae* A909 | NC_007432 | 2.83 | 3.63 | 3.14 |  | 80.57% | 90.53% | 85.71% |  |
| *Acinetobacter baumannii* ATCC 17978 | NC_009085 | 2.24 | 2.43 | 2.08 |  | 63.87% | 64.29% | 58.33% |  |
| *Agrobacterium fabrum* str. C58 | NC_003062 | 2.61 | 3.13 | 3.09 |  | 57.18% | 62.37% | 63.64% |  |
| *Brevundimonas subvibrioides* ATCC 15264 | NC_014375 | 2.95 | 3.67 | 2.62 |  | 53.04% | 51.41% | 65.52% |  |
| *Bacillus thuringiensis* BMB171 | NC_014171 | 2.55 | 3.44 | 2.17 |  | 81.02% | 86.96% | 83.33% |  |
| *Campylobacter jejuni* 81-176 | NC_008787 | 3.08 | 5.31 | 3.15 |  | 58.21% | 62.20% | 57.69% |  |
| *Francisella tularensis* Schu 4 | NC_006570 | 2.63 | 4.05 | 3.98 |  | 59.76% | 64.35% | 66.67% |  |
| *Streptococcus mutans* UA159 | NC_004350 | 2.82 | 3.97 | 2.33 |  | 82.25% | 92.86% | 83.33% |  |
| *Escherichia coli* O157:H7 EDL933 | NC_002655 | 3.05 | 3.67 | 2.88 |  | 55.77% | 63.18% | 62.22% |  |
| *Ralstonia solanacearum* GMI1000 | NC_003295 | 2.95 | 3.73 | 2.69 |  | 58.24% | 71.52% | 69.23% |  |
| *Streptococcus suis* P1/7 | NC_012925 | 2.83 | 3.26 | 2.48 |  | 80.15% | 90.84% | 76.00% |  |
| *Staphylococcus aureus* USA300_TCH1516 | NC_010079 | 2.93 | 3.82 | 2.26 |  | 77.64% | 90.80% | 96.77% |  |
| *Staphylococcus aureus* MW2 | NC_003923 | 2.98 | 3.71 | 2.22 |  | 76.29% | 94.05% | 96.30% |  |
| *Staphylococcus aureus* MSSA476 | NC_002953 | 2.96 | 3.86 | 2.27 |  | 75.88% | 94.32% | 96.97% |  |
| *Staphylococcus aureus* MRSA252 | NC_002952 | 2.90 | 3.91 | 2.27 |  | 75.96% | 93.10% | 96.97% |  |
| *Burkholderia cenocepacia* J2315 | NC_011000 | 2.82 | 3.44 | 2.84 |  | 53.88% | 61.02% | 44.00% |  |
| *Vibrio cholerae* O1 biovar eltor N16961 | NC_002505 | 2.87 | 4.81 | 2.81 |  | 58.35% | 71.43% | 77.78% |  |
| *Mycoplasma pneumoniae* M129 | NC_000912 | 2.60 | 5.74 | 3.11 |  | 77.14% | 79.25% | 66.67% |  |
| *Methanococcus maripaludis* S2 | NC_005791 | 2.73 | 3.29 | 3.05 |  | 52.76% | 49.06% | 45.61% |  |
| *Sulfolobus islandicus* M.16.4 | NC_012726 | 2.65 | 3.92 | 2.85 |  | - | - | - |  |
